# Synchronous cancers of gallbladder carcinoma and combined hepatocellular cholangiocarcinoma: an unusual case and literature review

**DOI:** 10.1186/s12885-018-4969-2

**Published:** 2018-10-29

**Authors:** Zhan-Guo Zhang, Yan Chen, Ran Ji, Ya-Jie Zhao, Jian Wang, Lily Robinson, Xiao-Ping Chen, Lei Zhang

**Affiliations:** 1 Hepatic Surgery Center, Institute of Hepato-Pancreato-Bililary Surgery, Tongji Hospital, Tongji Medical College, Huazhong University of Science and Technology, Wuhan, 430030 People’s Republic of China; 2 Department of Pediatrics, Union Hospital, Tongji Medical College, Huazhong University of Science and Technology, Wuhan, 430022 Hubei Province People’s Republic of China; 3 Hepatic Surgery Center, Tongji Hospital, Tongji Medical College, Huazhong University of Science and Technology, 1095 Jiefang Avenue, Wuhan, 430030 People’s Republic of China; 4 Department of Pediatrics, Pennsylvania State University College of Medicine, Hershey, PA 17033 USA; 5 Hepatic Surgery Center, Department of Surgery, Tongji Hospital, Tongji Medical College, Huazhong University of Science and Technology, Wuhan, 430030 People’s Republic of China

**Keywords:** Synchronous primary cancers, Gallbladder carcinoma, Combined hepatocellular cholangiocarcinoma, Diagnosis, Surgical treatment

## Abstract

**Background:**

Synchronous primary cancers in gallbladder and liver are rarely reported. Here we report an unusual case of synchronous cancers of gallbladder carcinoma and combined hepatocellular cholangiocarcinoma.

**Case presentation:**

Several lesions in the gallbladder and in adjacent parenchyma of liver were discovered in a 65-years-old woman by imaging examination. Surgical resection was performed following a diagnosis of primary gallbladder carcinoma with local hepatic metastasis. Histological examination confirmed the diagnosis of primary gallbladder carcinoma, and the lesions in the liver consisted of hepatocellular carcinoma simultaneously with cholangiocarcinoma. Adjuvant chemoradiation therapy was not performed due to the patient’s refusal of the treatment. Unfortunately, the patient died of widespread metastasis 8 months after the operation.

**Conclusions:**

The disease needed to be differentially diagnosed from gallbladder carcinoma with hepatic metastasis. Aggressive surgical approach should be based on a balance between the risk of surgery (morbidity and mortality) and the outcome.

## Background

With the widespread of regular medical check-ups and the improvement in early diagnosis, the occurrence of synchronous primary cancers (SPC) is becoming more frequent in the past decade [1]. However, the knowledge of the clinical features and outcomes of SPC remains limited. A high incidence of SPC has been found in the liver or gallbladder; nevertheless, SPC involving both liver and gallbladder are regarded as a rare occurrence, and only seven cases have been reported in English literature for the past seventy years. Here, we reported a case of synchronous double cancers of gallbladder carcinoma (GC) and combined hepatocellular cholangiocarcinoma (CHC).

## Case presentation

**Fig. 1:**
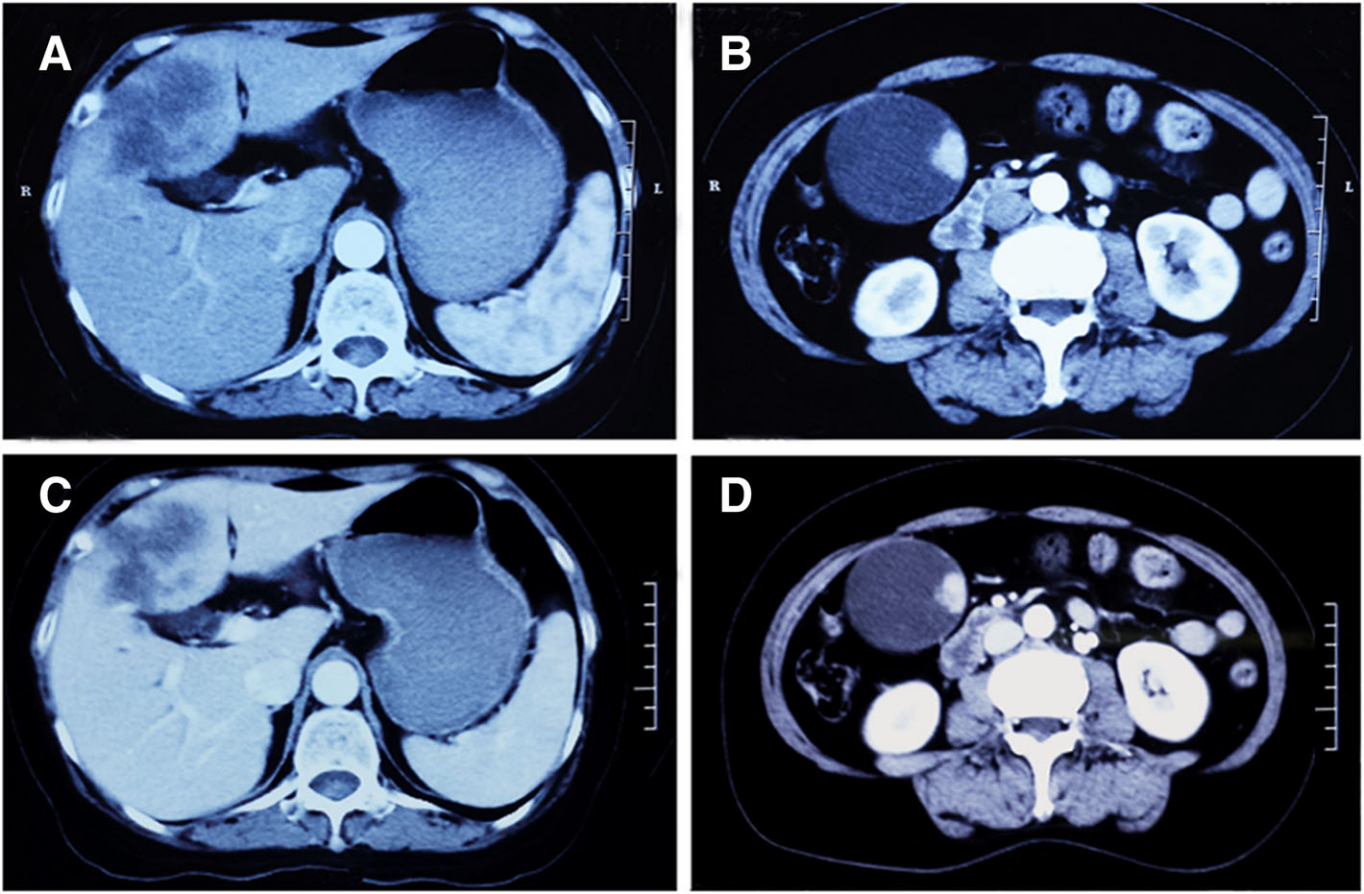
Abdominal multi-detector computed tomography-scan showing 3 × 3 cm mass in gallbladder and 6.2 × 4.5 cm lesion in segment IVb of the liver. **a** and **b** Artery phase, **c** and **d** Venous phase

**Fig. 2:**
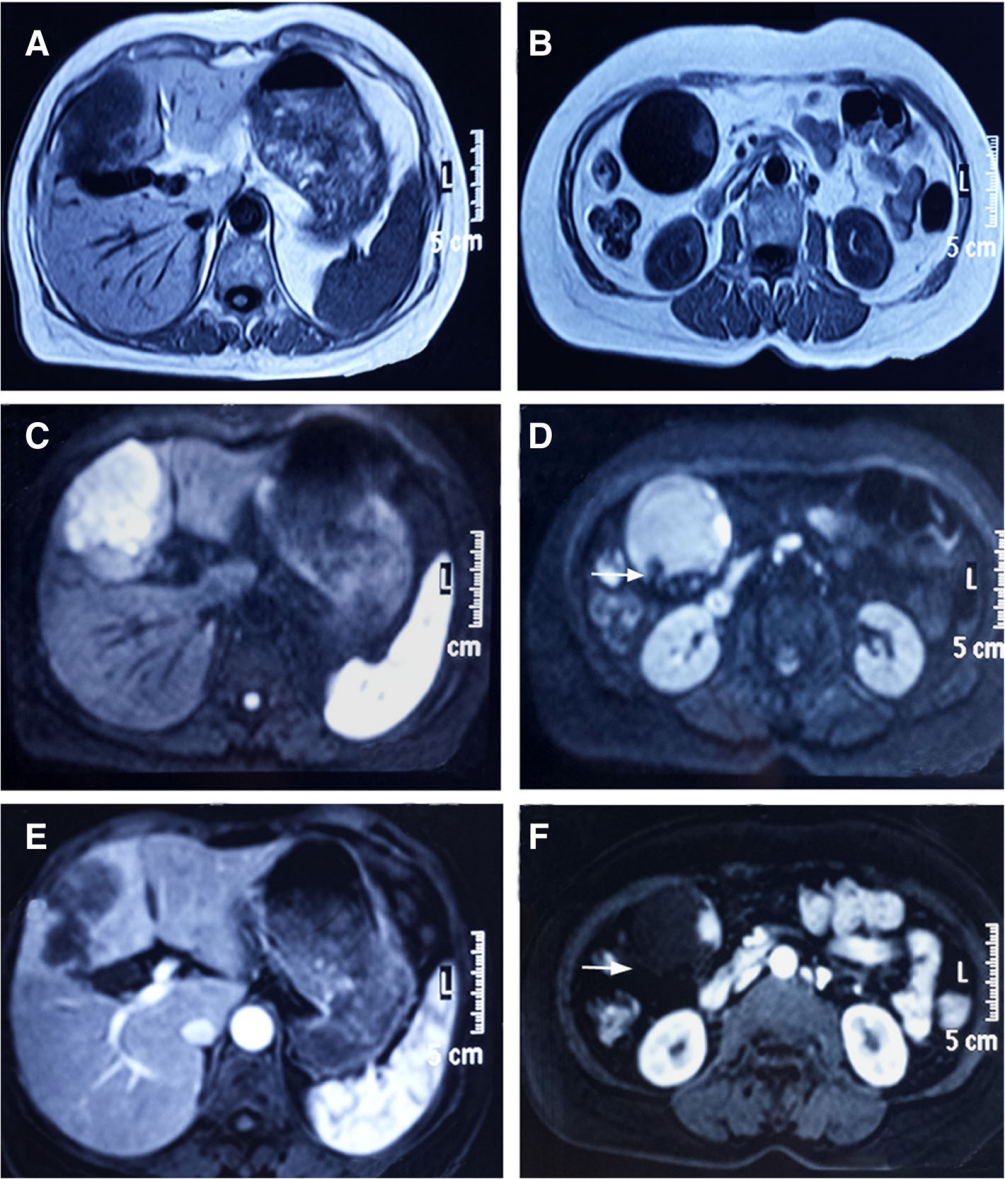
Abdominal magnetic resonance imaging-scan demonstrating gallbladder stones and 3 × 3 cm mass in gallbladder, and 6.2 × 4.5 cm lesion segment IVb of the liver. **a** and **b** T1-weighted images, **c** and **d** T2-weighted images, **e** and **f** Artery phase, **d** and **f**, arrow: Gallbladder stones

A 65-years-old female Chinese woman was admitted to our hospital with the chief complaint of abdominal pain in the right upper quadrant for the past 20 days. There was no remarkable family, medical or genetic history. The patient was in good general health and had no significant weight loss. Her vital signs (including heart rate, respiration rate, blood pressure and body temperature) were within normal limit. There were two positive signs during the physical examination, anemic conjunctiva and tenderness in the right upper quadrant. Complete blood count and serum biochemistry data on admission remained normal except hemoglobin, 9.5 g/dl. Significant abnormalities were found in the tumor marker, demonstrated by a normal serum level of alpha-fetoprotein (AFP; 4.85 ng/ml, normal: 0–8.78 ng/ml) and elevated levels of carcinoembryonic antigen (CEA; 16.3 ng/ml, normal: 0.5–5.0 ng/ml), carbohydrate antigen125 (CA125; 371.2 U/ml, normal: 1–35 U/ml) and CA19–9 (358.96 U/ml, normal: 2–37 U/ml). Multi-detector computed tomography (CT) scan of the abdomen showed distension of the gallbladder with gallbladder stones and several homogeneous high-density masses in the gallbladder fundus (intense enhancement on artery and portal venous phase, low attenuation on delayed phase), and multiple hypodensity tumorous lesions adjacent to the gallbladder (mild irregular enhancement at the periphery of the lesions on artery and portal venous phase, further enhancement on delayed phase), which were located in the lower part of segment IV of the liver (Fig.1a-d). Magnetic resonance imaging (MRI) with perfusion-weighted imaging confirmed the presence of gallbladder stones and solitary 3 × 3 cm enhanced lesions in the gallbladder, and 6.2 × 4.5 cm hypovascular tumors in the liver (Fig. 2a-f). The data of abdominal ultrasonography was consistent with the above data. Thus, the preoperative diagnosis was GC with hepatic metastasis.

The patient was informed of the risks involved with the surgery before consent for the operation was obtained. After sufficient preoperative preparation, the patient underwent an exploratory laparotomy. During laparotomy, the gallbladder was enlarged to 16 × 6 × 6 cm and showed wall thickening (the thickness was 1 cm). There was a palpable mass felt on the surface of the gallbladder fundus portion. Exploration also showed an 8 × 6 cm rigid lesion fused by multiple masses in liver segment IVb and V and a 1 × 1 cm lesion in segment VIII. Moreover, sporadic lesions on the diaphragm and enlarged station 8 lymph nodes were seen. The patient underwent cholecystectomy, resection of liver segment V, of the lower part of segment IV and partial segment VIII, regional lymphadenectomy and resection of lesions on diaphragm.

**Fig. 3:**
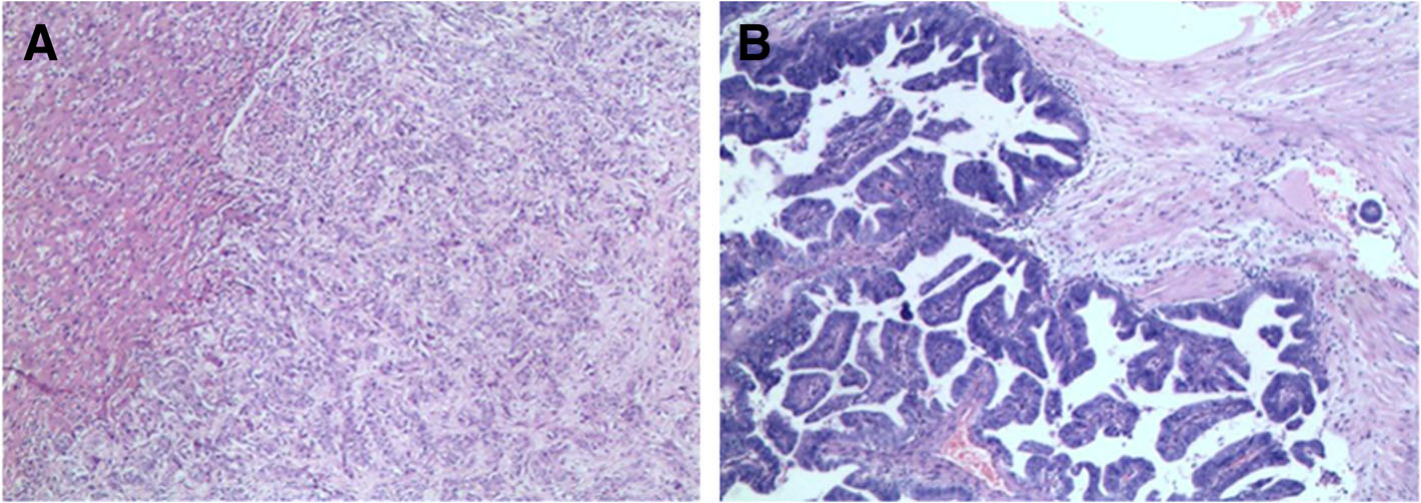
Histopathological examination of resected specimen (Hematoxylin–eosin staining, magnification× 100). **a** Combined hepatocellular cholangiocarcinoma in segment IVb of liver, **b** Well-differentiated papillary adenocarcinoma invading the muscularis propria

The post-operative histopathological examination revealed synchronous double cancers in the liver and gallbladder, which were GC (well-differentiated papillary adenocarcinoma invading the muscularis propria) and CHC (Fig. 3a and b). The examination also showed that the metastases in lymph nodes and diaphragm were both from CHC in the liver.

After 10 days of recovery, the patient was discharged without complications. Adjuvant chemoradiation therapy was not performed due to the patient’s refusal. Unfortunately, the patient died of widespread metastasis 8 months after the operation.

## Discussion and conclusions

The incidence of multiple primary cancers (MPC) has been reported to vary from 0.7 to 11.7% [2]. The diagnostic criteria have by adopted by most clinicians for MPC: 1) each tumor must be malignant determined by histological evaluation; 2) the tumors must be separate and distinct; 3) the probability that one is a metastasis from the other tumor must be ruled out [2, 3]. According to these criteria, this case was determined to be double SPC. A high incidence of synchronous primary cancers has been found in liver. Lee JH et al. reported that the most common synchronous sites were colorectal cancer (37.2%), followed by lung cancer (18.6%), esophageal cancer (16.8%), liver cancer (9.7%), kidney cancer (4.4%) and gastric cancer (3.4%) [4]. A study by Zeng QA et al. showed that the incidence of synchronous cancer in liver cancer patients was 3.4% [1]. SPC rarely occurs in the gallbladder (14 cases total). SPC in both of the above organs is rarely reported. Due to the lack of apparent radiological features, this kind of SPC is not easily differentiated from hepatic metastasis of gallbladder carcinoma. There are 7 cases reported in the English language so far [5–11]. The results can be seen in Table 1.

**Table 1:** Literature Review of Synchronous Primary Cancers Occurring in Gallbladder and Liver

Cases	Species	Age(y)/ Sex	Risk of liver tumor	Gallbladder stones	Location of tumor in liver	Histopathological examination	Treatment	Prognosis (Months)
Imada J et al [5]	Human	70/F	HCV/Cirrhosis	No	SVII	HCC/CCD/GC^w^	Conservative treatment	4/Die of Liver failure
Taniai N et al [6]	Human	83/F	N/A	With	SVIII	ICC^w^/GC	Laparotomy	N/A
La Greca G et al [7]	Human	N/A	N/A	With	SIV	HCC^w^/GC	Laparotomy	N/A
Kin JW et al [8]	Human	63/M	HBV/Cirrhosis	With	SIII	HCC/GC^m^	Laparotomy	17/Alive
Unver M et al [9]	Human	65/M	HB*V*/*w*ithout cirrhosis	With	SI*V*/VII	HCC/GC^w^	Laparotomy	6/Alive
Lu J et al [10]	Human	67/F	No	No	SIV/V	HNC/GC	Laparotomy	N/A
Jakab C et al [11]	*Pogona vitticeps*	N/A	N/A	No	Peripheral part	ICC^w^/GC^w^/GA	Laparotomy	Euthanasia

*M* male, *F* female, *S* segment, *HCC* hepatocellular carcinoma, *GC* gallbladder adenocarcinoma, *CCD* adenocarcinoma of the common bile duct, *HNC* hepatic neuroendocrine carcinoma, *w* well differentiated, *m* moderately differentiated, *ICC* intrahepatic cholangiocellular carcinoma, *GA* gallbladder adenoma

**Fig. 4:**
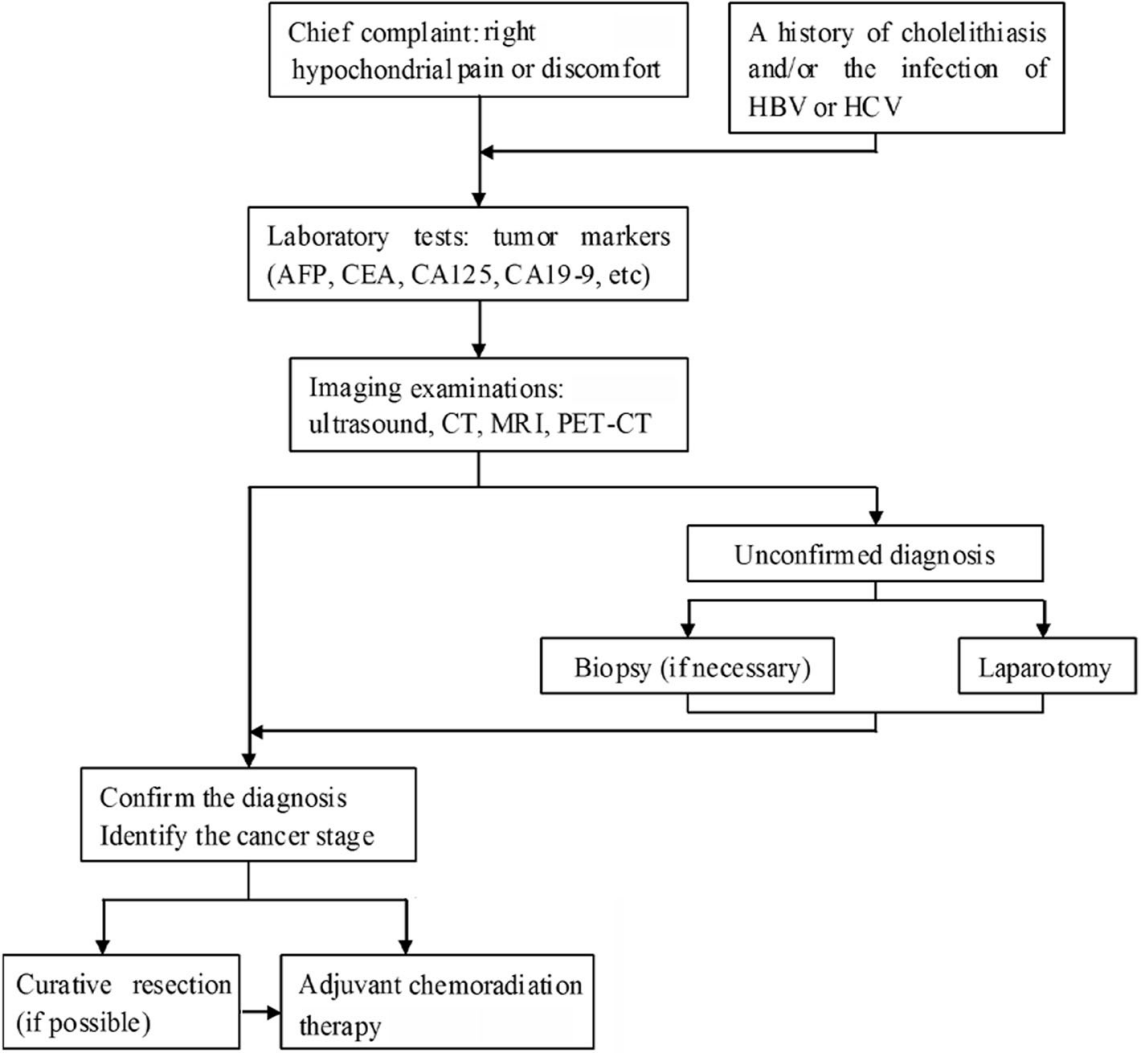
Clinical algorithms for the evaluation of synchronous primary cancers in liver and gallbladder. AFP = alpha-fetoprotein, CEA = carcinoembryonic antigen, CA125 = carbohydrate antigen125, CT = computed tomography, MRI = magnetic resonance imaging, PET-CT = positron emission computerized tomography

In this series of 7 cases reviewed in literature, only one case was correctly diagnosed before operation (GC and intrahepatic bile duct carcinoma). One case was diagnosed as GC and hepatocellular carcinoma by intraoperative frozen section. The detailed diagnostic procedure is shown in Fig. 4. CT and MRI are considered the best diagnostic techniques for cancer staging and surgical strategy guiding. Although the patient accepted the CT and MRI examinations, we did not take SPC of GC and CHC as a possible diagnosis before operation. The reasons may be: 1) low incidence and lack of experience; 2) lack of obvious symptoms and typical radiological features: both metastatic lesions of GC and CHC manifested hypovascular lesions in the liver; 3) non-specific tumor markers. Most of patients with CHC had elevated serum level of AFP [12]. The case presented here had normal level of AFP and elevated serum levels of CEA, CA125 and CA19–9. These elevated tumor markers were commonly detected in patients with GC. We searched literatures [13–16] and listed the main differences between SPC and GC with hepatic metastasis in Table 2. Recently, the studies showed that positron emission computed tomography (PET) scan was useful in differentiating malignant from benign disease, in preoperative staging, and detecting postoperative residual disease [13–17]. Histopathology from biopsy or liver resection remains the golden diagnostic standard [1]. If the lesions can be surgically resected, histological examination of the whole lesion is more reliable than a biopsy, because insufficient, missed or ample necrotic tissue may not be representative of the entire lesion [18]. Needle-tract implantation and complications (such as prolonged internal bleeding, bile leakage and infection) are other reasons that preoperative biopsy of the liver is not recommended [19].

**Table 2:** Differential Diagnosis Between SPC and GC with HM

	SPC	GC with HM
Incidences	Rare, 8 cases	Geography/ethnicity (1.1~ 27/10^5^)
Gender	Major in F (F/M = 2/1)	Major in F (F/M = 2~ 6/1)
Age(yrs)	Old age (Mean = 68.8)	Old age (Mean = 67)
HBV/HCV	Partial patients	Not
Gallbladder stones	50%	~ 85%
AFP	Elevated in partial patients	Not
CEA	Elevated	Elevated
CA19–9	Elevated	Elevated
Number of liver masses	Simple or multiple	Multiple
Location of liver masses	Any segments in the liver	Adjacent to the gallbladder
Invasion of GC	Not found	Presentation
Imaging manifestation	Hypovascular or hypervascular	Hypovascular

*SPC* synchronous primary cancers, *GC* gallbladder adenocarcinoma, *HM* hepatic metastasis, *M* male, *F* female, *HBV* hepatitis B virus, *HCV* hepatitis C virus, *AFP* alpha-fetoprotein, *CEA* carcinoembryonic antigen, *CA19–9* carbohydrate antigen 19–9

Curative resection, if possible, most effectively prolongs patient survival. However, in cases with SPC in the liver and gallbladder, the choice of treatment strategy should be made carefully in conjunction with the treatment for the second malignancy. There are few reports on how to treat patients with this rare disease, which remains a key challenge. The patient presented here underwent laparotomy, which included cholecystectomy, resection of liver segment V, of the lower part of segment IV and partial segment VIII, regional lymphadenectomy and resection of lesions on diaphragm. Unfortunately, the patient died of widespread metastasis 8 months after the operation. Reviewing the literature, the longest survival of 17 months happened in the patient with PC and hepatocellular carcinoma.

The pathogenesis of SPC has not been clarified, some factors such as genetic factors, hormones, environmental carcinogens, dietary factors, infective agents, previous therapy, alcohol and smoking are involved [20, 21]. Gallbladder stones and/or infectious agents develop cancer as a result of recurrent trauma and chronic inflammation [8]. One reasonable hypothesis focuses on chronic irritation of the mucosa (e.g., from the physical presence of the stones and/or superimposed chronic infection such as from *Salmonella typhi*) leading to dysplasia (perhaps abetted by mutagenic secondary bile acids) and terminating in malignant change [22]. CHC is a rare tumor, accounting for 1.3% of primary hepatic malignancies [23]. Studies show that many etiologies, including the infection of hepatitis B and C virus, allelic losses, and divergent differentiation of hepatic stem/progenitor cells were associated with CHC [24–26]. Our previous study also suggested that CHC might arise from hepatic progenitor cells [27]. Otherwise, many studies manifest that chronic inflammation has been suggested as an important step to hepatocarcinogenesis [28, 29]. In this case, we speculate that chronic infection of gallbladder affects surrounding liver tissues, leading to inflammation of surrounding liver tissues, which was demonstrated by enhancement of segment IV during arterial phase (Fig. 2e). Liver inflammation promotes aberrant self-renewal and irritates malignant transformation of hepatic progenitor cells. Because of the rarity of SPC in the gallbladder and liver, clinical data is limited. So the detailed etiology and mechanism still need further clarification.

In conclusion, synchronous primary cancers involving both the liver and gallbladder are regarded as a rare occurrence. Due to the lack of apparent radiological features, it is not easily differentiated from hepatic metastasis of GC. It is necessary to develop more accurate diagnostic techniques and administer more refined treatment strategies to correctly diagnose SPC.

## Abbreviations

AFP: alpha-fetoprotein
CA125: carbohydrate antigen125
CEA: carcinoembryonic antigen
CHC: combined hepatocellular cholangiocarcinoma
CT: computed tomography
GC: Gallbladder carcinoma
MRI: magnetic resonance imaging
PET: positron emission computed tomography
SPC: synchronous primary cancers
